# Perception of pain during intravitreal injections: a clinical trial on the effect of entry site distance from the limbus on perceived pain

**DOI:** 10.1186/s40942-025-00769-z

**Published:** 2025-12-22

**Authors:** Janet Fan, Rodney Guiseppi, Biai Digbeu, Touka Banaee

**Affiliations:** 1 School of Medicine, The University of Texas Medical Branch, Galveston, TX USA; 2 Department of Ophthalmology and Visual Sciences, The University of Texas Medical Branch, 301 University Blvd, Galveston, TX 77550 USA; 3 Department of Biostatistics and Data Science, The University of Texas Medical Branch, Galveston, TX USA

**Keywords:** Anti-VEGF, Entry site, Intravitreal injections, Injection site, Limbus, Location, Pain perception, Visual analogue scale

## Abstract

**Purpose:**

Intravitreal anti-vascular endothelial growth factor injections have become increasingly common over the last decade. Pain perception during these injections varies based on factors such as injection location, type of anesthetic, needle gauge, medication used, etc. However, no previous studies have evaluated pain perception at different injection locations in relation to the limbus. Enhancing patient experience during these procedures may improve long-term compliance to treatment. Thus, this study aimed to evaluate the relationship between pain perception and injection sites at varying distances from the limbus.

**Methods:**

This prospective, randomized, single-blinded, single-center study examined pain perception in patients receiving intravitreal injections for various ocular conditions. Each patient received two injections at either 3.5 or 4.0 mm from the limbus in the inferotemporal quadrant. The order of injection location was randomized, and patients were blinded to the injection site. Pain was assessed immediately after each injection using a visual analogue scale from 0 to 10. A paired-sample t-test was used to compare pain perception between injection sites, and a repeated measures multivariable linear regression model was applied to predict the effects of other variables on pain perception.

**Results:**

A total of 53 patients were enrolled, receiving two injections. The overall mean pain score for all injections was 1.82 ± 1.64, ranging from 0 to 7. Injections administered 3.5 mm away from limbus had a slightly increased mean pain score (1.92 ± 1.64) compared to those at 4.0 mm away (1.72 ± 1.65), but this difference was not statistically significant (*p* = 0.45). Additional factors, including injection site location, age, gender, ethnicity, diabetic indications, number of prior injections, type of anesthesia, and medication type were not significant predictors of pain score during injections.

**Conclusion:**

Pain perception did not significantly differ between injections administered 3.5 mm and 4.0 mm away from the limbus. This study is the first to evaluate pain perception at varying injection distances relative to the limbus. Injection placement should be guided by physician preference and experience.

**Trial registration:**

This study was retrospectively registered on ClinicalTrials.gov on November 28, 2022 (ClinicalTrials.gov ID is NCT05640895).

## Background

Intravitreal anti-vascular endothelial growth factor (anti-VEGF) injections have become increasingly prevalent over the last decade, with over 100,000 intravitreal injections (IVI) performed monthly in the United States [1]. Some examples of anti-VEGF medications include ranibizumab, aflibercept, faricimab-svoa, and bevacizumab. The injections are routinely administered for the management of various ocular conditions, including diabetic retinopathy (DR), diabetic macular edema (DME), age-related macular degeneration (AMD), and retinal vein occlusion (RVO), etc [2–7]. Typically, treatment of these ocular diseases requires repeat injections to maintain therapeutic effect [6, 8, 9]. Given the frequent use of intravitreal injections, it’s important to optimize patient comfort and reduce patient pain and anxiety caused by the procedure. Moreover, pain with eye injections can cause unexpected and sudden eye movement, potentially leading to ocular complications and possible refusal of future treatment [10]. Efforts should be made to enhance the patient’s injection experience, which could positively impact long-term patient compliance.

Standard practice to reduce patient discomfort and pain includes the use of local anesthesia prior to injection [11]. Several established techniques used for local anesthesia include subconjunctival administration of lidocaine, application of lidocaine via a cotton tip applicator onto the surface of the conjunctiva and sclera, application of lidocaine gel, or applying topical drops such as proparacaine or tetracaine onto the surface of the eye [12]. However, systematic review showed that with moderate level of evidence, no single anesthetic technique could be defined as the best option for intravitreal injections [12]. 

More studies have also been conducted to evaluate what other factors are associated with increased pain and worse intravitreal injection experience. Some modifiable factors that have been examined include injection needle sizes, type of anti-VEGF medications, gender, number of previous injections, and varying injection location by quadrants of the globe [13–16]. A meta-analysis, consisting of 9 studies and 998 eyes, has shown that thinner needles (higher gauge) are associated with less pain [13]. There is also conflicting data regarding the perception of pain when comparing different anti-VEGF agents, with one study showing that there was no significant difference in pain scores between aflibercept, ranibizumab, or a dexamethasone implant [14], or bevacizumab, bevacizumab/kenalog, or ranbizumab,^17^ while another reported that aflibercept had higher pain scores compared to ranibizumab [18]. Another study by Inaltekin et al. [15] reported that bevacizumab was more painful than both aflibercept and ranibizumab. Additionally, other studies have also shown varied effects of gender on pain, with some reporting that females experience higher [16], lower [17], or no difference [19] in pain compared to males. There is also conflicting data on whether quadrant location impacts pain severity, with some studies showing no correlation [19, 20] while others providing support for this association [1]. 

However, to our knowledge there is no literature evaluating pain perception at different injection sites in relation to the limbus. Current standard practice entails placing intravitreal injections at the pars plana, specifically anywhere from 3.0 to 3.5 mm posterior to the limbus for an aphakic or pseudophakic eye, and 3.5 to 4.0 mm posterior to the limbus for a phakic eye [11, 21]. Quadrant selection is also based on physician preference and patient consideration. Therefore, this prospective study aimed to investigate the relationship between pain perception and location of intravitreal injection placement at either 3.5 or 4.0 mm away from the limbus. We hypothesized that there would be increased pain at the 3.5 mm location compared to the 4.0 mm location. Anatomically, the shape of the ciliary body is thicker anteriorly near the lens and then gradually thins as it tapers down posteriorly toward the retina. Therefore, injections placed more anteriorly may pierce thicker and thus more densely innervated ciliary tissue, leading to increased pain sensation.

## Methods

### Study design and patients

This was a prospective, randomized, single-blinded, and single center study on pain perception in patients receiving intravitreal injections for various pathologic eye conditions. This study was approved by the Institutional Review Board of the University of Texas Medical Branch (IRB number 22–0228). The study was conducted in adherence to the ethical principles originating from the Declaration of Helsinki and the Health Insurance Portability and Accountability Act. This study was also registered on November 28, 2022 with ClinicalTrials.gov (ClinicalTrials.gov ID: NCT05640895).

Eligible patients were English speaking patients aged 18 years and older, pseudophakic, had a frequent need for intravitreal anti-VEGF injections (e.g. ranibizumab, aflibercept, bevacizumab, or faricimab-svoa), and had an indication to be treated for age-related macular degeneration (AMD), diabetic macular edema (DME), choroidal neovascular membranes (CNVM), vitreous hemorrhage (VH) due to proliferative diabetic retinopathy (PDR), retinal vein occlusion (RVO), or cystoid macular edema (CME). Exclusion criteria included those under 18 years of age, no prior intravitreal injection experience, recent ocular surgery, need for ocular surgery in the near future, presence of natural crystalline lens, active ocular inflammation, osteogenesis imperfecta and cognitive disability. All patients provided written informed consent to participate in the study.

Recruitment of patients was performed daily during retina clinic from November 14, 2022- to February 26, 2024. Once patients met the inclusion criteria, the details of the study were discussed with the patient and consent was obtained before participation in the study. Patients were then assigned a random number from a randomization chart. If they were assigned an even number, they were given their first injection 3.5 mm from the limbus and those who were assigned an odd number were given their first injection at 4.0 mm from the limbus. At their next injection appointment, they were given the second injection at the other location (i.e. those who got their first injection at 3.5 mm from the limbus were given their next injection at 4.0 mm from the limbus and vice versa). Patients were also blinded as to which location they would be injected at each time. In patients who required injections in both eyes and met the inclusion criteria for both eyes, only one eye was randomly selected to be included in the study. The primary outcome of the study was to assess the difference in the perception of pain at 3.5 mm vs. 4 mm from the limbus.

### Injection procedure

For each injection, we first applied a drop of topical proparacaine, followed by application of 5% betadine, and then an anesthetic agent. Two methods of analgesia were used in the study: some patients were administrated lidocaine gel 3.5% on the surface of the injection site for 5 min and some patients were administered 2 sequential cotton tip applicators soaked in lidocaine 4% onto the injection site for a total of 2 min. The anesthetic used during the first injection administration was repeated at the second injection administration to maintain consistency in anesthesia technique for each patient. Following the administration of anesthesia, a lid speculum was placed, and another drop of betadine was instilled on the ocular surface. Using a caliper, the distance from the limbus was measured in the inferotemporal quadrant at either 3.5 or 4.0 mm, and a small indentation at the location was made using the tip of the caliper. For patients undergoing anesthesia with a cotton tipped applicator, the injection was given at the site of placement of the applicator. Anti-VEGF medication was then administered using a 32-gauge needle (Steriject, TSK Lab Intl. Tochigi-Ken, Japan) at the injection site, which was easily discernible due to pooling of betadine at the site marked by the calipers. Anti-VEGF medications used in this study included bevacizumab (Avastin, Genentech, 1 DNA Way Mailstop, South San Francisco, CA 94080), faricimab-svoa (Vabysmo, Genentech, 1 DNA Way Mailstop, South San Francisco, CA 94080), or aflibercept (Eylea, Regeneron, 777 Old Saw Mill River Road, Tarrytown, NY 10591). All intravitreal injections were performed by a single surgeon in the inferotemporal quadrant. Visual acuity and IOP were checked after the procedure.

### Data collection

**Fig. 1:**
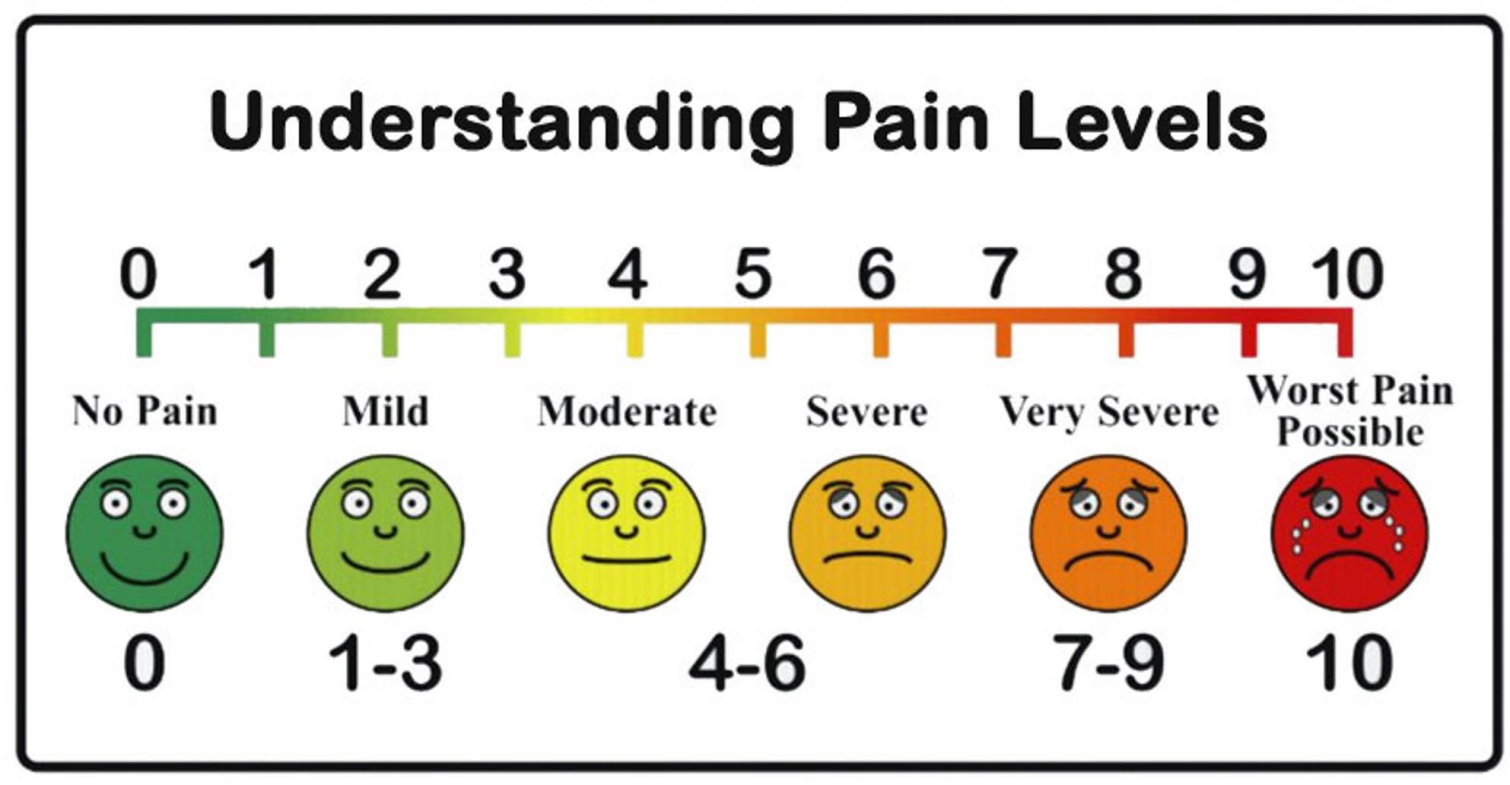
Visual analogue scale for pain used by patients in this study

Immediately after each injection, patients were asked to rate their pain from 0 to 10 using a visual analogue pain scale. A score of 0 represents no pain and a 10 represents the worst pain possible. The scale is also illustrated with face emojis corresponding to a written description of pain perception, ranging from no perception of pain which is represented by a green, fully smiling face to perception of the worst pain possible, which is represented by a red, crying, fully frowning face (Fig. 1). The visual analogue pain scale has been shown to be reliable and a frequently used pain assessment tool employed in various prior ophthalmological studies [17, 22–24]. Patients were also specifically informed to assess their pain from the injection itself and not from any irritation or pain before or after the injection.

### Statistical analysis

All data was entered into Microsoft Excel and data analysis was conducted in R version 4.3.2 and SAS version 9.4 (SAS Institute Inc. North Carolina, USA). A power analysis was performed to determine the minimum sample size required to detect a statistically significant difference in pain perception between the two injection sites. The power analysis was based on an alpha level of 0.05, a desired power of 0.80, and effect size of 0.5. Based on this analysis, we determined 33.4 participants would be needed to achieve adequate statistical power for the primary outcome. Patient characteristics were summarized using descriptive statistics. A paired-sample t-test was used to compare pain perception between injection sites 3.5 mm and 4.0 mm away from the limbus. A paired-sample t-test was conducted to assess differences within the study population subgroups, including gender, ethnicity, indication, anesthesia type, medication, and injection history. Further analysis was performed using a repeated measures multivariable linear regression model to predict the effects of multiple variables on pain score. Statistical significance accepted for the study was < 0.05.

## Results

### Demographics and baseline patient characteristics

A total of 53 patients were enrolled and completed both injections. Baseline demographic data and injection characteristics are summarized in Table 1. The trial consisted of 21 (39.62%) male participants and 32 (60.38%) female participants, and the average age of all participants was 74.75 years. Their ages ranged from 46 to 95 years. The majority were White (*n* = 36), followed by Hispanic (*n* = 12), and then Black (*n* = 5). The majority of participants had a previous history of receiving greater than 3 injections (*n* = 45), while 8 participants received 1–3 injections prior to this study.

**Table 1:** Baseline patient and injection characteristics

Patient data		*N* (%)
**Age**		
Mean, Range (years)	74.75, 46–95	
**Gender**		
Female	32 (60.38)	
Male	21 (39.62)	
**Ethnicity**		
White	36 (67.92)	
Hispanic	12 (22.64)	
African American	5 (9.43)	
**Previous Injection History**		
1–3	8 (15.09)	
More than 3	45 (84.91)	
**Indication**		
ARMD	27 (50.94)	
DME	16 (30.19)	
VH secondary to PDR	4 (7.54)	
Central RVO/Branch RVO	3 (5.66)	
CNVM	2 (3.77)	
CME	1 (1.89)	
**Indications**		
Diabetic (VH secondary to PDR and DME)	20 (37.74)	
Non-diabetic (ARMD, BRVO, CME, CNVM, and CRVO)	33 (62.26)	
**Medication Injection 3.5 mm**		
Bevacizumab	27 (40.3)	
Aflibercept	21 (51)	
Faricimab-svoa	5 (0.09)	
**Medication Injection 4 mm**		
Bevacizumab	28 (52.83)	
Aflibercept	21 (39.62)	
Faricimab-svoa	4 (7.55)	

### Treatment indication and characteristics

All 53 patients received two intravitreal injections of anti-VEGF in the same eye at an injection site 3.5 mm and 4.0 mm away from the limbus. The main indication for intravitreal anti-VEGF injections was ARMD (*n* = 27), followed by DME (*n* = 16), and VH secondary to PDR (*n* = 4). The least common indications included central RVO (*n* = 2), branch RVO (*n* = 1), CNVM (*n* = 2), and CME (*n* = 1). The most frequently administered medications were bevacizumab, aflibercept, and then faricimab-svoa from highest to lowest frequency.

### Location

Overall, the mean pain score for all injections regardless of location was 1.82 ± 1.64 and scores ranged from 0 to 7. When separated based on injection site location, those who received the injection 3.5 mm away from the limbus had an increased mean pain score of 1.92 ± 1.64 compared to those who received it at 4.0 mm away from the limbus with a mean pain score of 1.72 ± 1.65. However, when analyzed with a paired t-test this difference was not found to be statistically significant (t(52) = 0.77, *p* = 0.45), indicating that there was no significant difference in pain experienced between the two injection sites (Table 2). Additionally, Table 2 presents a subgroup analysis of pain scores based on gender, anesthesia type, medication type, indication, and history of injection.

**Table 2:** Paired T-tests comparing the mean pain score by Anti-VEGF injection location among patients

	Anti-VEGF Injection Location			
	3.5 mm	4.0 mm	Mean difference (Standard deviation of the differences)	*P*- value
Overall mean pain score (*n* = 53)	1.92 ± 1.64	1.72 ± 1.65	0.21 (1.96)	0.445
**Gender**				
Female (*n* = 32)	1.78 ± 1.60	1.56 ± 1.78	0.22 (2.15)	0.569
Male (*n* = 21)	2.14 ± 1.71	1.95 ± 1.43	0.19 (1.69)	0.612
**Anesthesia Type**				
Lidocaine 4% on cotton tip applicator (*n* = 35)	1.80 ± 1.62	1.86 ± 1.48	-0.06 (1.45)	0.818
Lidocaine 3.5% gel (*n* = 18)	2.17 ± 1.69	1.44 ± 1.95	0.72 (2.67)	0.268
**Indication**				
Diabetes related (*n* = 20)	1.95 ± 1.82	1.70 ± 1.84	0.25 (2.17)	0.613
Non-diabetes related (*n* = 33)	1.91 ± 1.55	1.73 ± 1.55	0.18 (1.86)	0.579
**Medication Type** ^*^				
Aflibercept (*n* = 19)	1.79 ± 1.51	1.79 ± 2.04	0.00 (2.19)	1.000
Bevacizumab (*n* = 26)	2.19 ± 1.74	1.73 ± 1.31	0.46 (1.68)	0.173
Faricimab-svoa (*n* = 4)	1.00 ± 0.82	1.50 ± 1.91	-0.50 (1.29)	0.495
**Number of previous injections**				
1–3 injections (*n* = 8)	2.13 ± 2.47	1.75 ± 1.16	0.38 (1.69)	0.549
>3 injections (*n* = 45)	1.89 ± 1.48	1.71 ± 1.73	0.18 (2.03)	0.559

^*^Four patients were excluded from this analysis due to usage of 2 different anti-VEGF medications between injections

### Comprehensive analysis of pain predictors

A repeated subjects’ multivariable linear regression model analysis was further conducted to evaluate how well injection site location, age, gender, ethnicity, diabetic indications, number of prior injections, type of anesthesia, and medication type can predict pain scores during injection with anti-VEGF therapy. However, results show that none of these variables were statistically significant predictors of mean pain score during injections of anti-VEGF (Table 3).

**Table 3:** Repeated subjects multivariable linear regression model predicting the effects of variables of interest on mean pain score

Variable	Estimate (β)	95% Confidence interval			*P*-value
Location (4 mm vs. 3.5 mm [Reference])	-0.11		-0.38	0.15	0.4004
Age	0.01		-0.01	0.04	0.3953
Gender (Males vs. Females [Reference])	0.21		-0.16	0.58	0.2594
**Ethnicity**,** Reference = White**					
African American	-0.19		-0.92	0.53	0.5965
Hispanic	0.12		-0.59	0.84	0.7332
Indication (Diabetic vs. Non-diabetic [reference])	0.14		-0.42	0.70	0.6290
Number of prior injection (1–3 vs. More than 3 [Reference])	0.08		-0.46	0.61	0.7786
Type of Anesthesia (Topical Lidocaine Gel 3.5% vs. Topical Lidocaine 4% [Reference])	-0.11		-0.51	0.29	0.5985
**Medication Type**,** Reference = Bevacizumab**					
Aflibercept	-0.16		-0.62	0.31	0.5121
Faricimab- svoa	-0.27		-1.15	0.61	0.5479

## Discussion

Intravitreal injections with anti-VEGF agents are the primary treatment option for a variety of retinal diseases and are one of the most performed ophthalmic procedures worldwide [1]. However, they can also cause patients stress and anxiety due to the anticipation of receiving an intravitreal injection. Gualino et al. [25] conducted a survey study, including 904 patients, on their experience receiving anti-VEGF therapy for their macular diseases. They were asked to rate their stress on a scale of 1–10, and the study revealed that 30% of patients had stress levels between 6 and 10, and 8% reported a maximum stress level of 10 [25]. Another study by Tailor et al. [26] specifically examined patient experiences at different stages of the intravitreal injection process. A total of 42 patients were administered a questionnaire and asked to rate their feelings of distress at each step of the injection using a visual analogue scale between 1 and 10. They considered a score of 4 to be significantly uncomfortable, and they found that the most unpleasant steps of the procedure for patients included waiting for the injection, application of the drape, insertion of the speculum, and needle entry, which had the highest mean score of 3.2. Needle entry also had the highest frequency (19%) of scores graded higher than 4 [26]. These results are also supported by the findings from a systematic review conducted by Senra et al. [27] on the psychological impact of anti-VEGF treatments for wet ARMD, which included a literature analysis of 14 papers. Common findings from the review emphasized that patients often experience discomfort and fear during anti-VEGF injections, specifically during needle entry, application of drops, insertion of speculum, waiting for the injection, experiences of pain, fear of losing sight and of the unknown, and experiencing unwanted side effects [27]. Additionally, another study by Polat et al. [28] was conducted to determine factors that influence compliance to anti-VEGF therapy in patients with AMD. They interviewed 314 patients who received ranibizumab for treatment by phone and found that 232 failed to comply with regular follow up for subsequent doses. The most common reason for non-compliance was that patients were afraid of intravitreal injections in 29.6% of patients [28]. Since intravitreal injections are frequently indicated for numerous diseases and require repeat treatment to have therapeutic effect, it is of paramount importance to identify modifiable factors that can help reduce anxiety and discomfort and alleviate pain experienced by patients to promote compliance with future therapy.

Despite their widespread use, the technique for administering intravitreal injections varies significantly amongst providers based on previous training and preference. There are general injection guidelines, but there is no consensus on the optimal injection technique that minimizes patients’ pain, given the various options available for local anesthesia, type of anti-VEGF medications, injection site location, etc [11, 21]. Furthermore, there are limited studies examining the effect of injection location on pain perception. A few studies have examined injection site location and pain based on quadrant injection location. Karimi et al.^1^ conducted a prospective, randomized, clinical trial that examined 1004 eyes from 1004 patients, who were assigned to receive injections in four different quadrants. Patients rated their pain based on a visual analogue pain scale of 0–10. They found that pain was significantly correlated with injection site, with patients experiencing the most pain at the superotemporal quadrant and the least pain at the superonasal quadrant [1]. On the other hand, Doguizi et al. [19] conducted a prospective, single-center study, examining factors contributing to pain with intravitreal injections including 119 patients, who received intravitreal aflibercept in the superotemporal or inferotemporal quadrant. Patients reported their pain using a visual analogue scale, and results showed that pain was not significantly correlated with quadrant location (*p* = 0.842) [19]. Moisseiev et al. [20] also conducted a similar study consisting of 218 patients, who received intravitreal bevacizumab in all four quadrants and found that pain did not correlate with location. However, there was a trend toward decreased pain in inferior locations, but this was also not found to be significant (*p* = 0.065) [20].

Besides quadrant specifications, there were no other studies in the literature examining the relationship between pain perception and injection site location in relation to the limbus. Thus, this study was designed to assess the perception of pain from intravitreal injections at two different locations in relation to the limbus, while keeping the quadrant for injection consistent. We hypothesized that patients would experience increased pain from injections administered 3.5 mm away compared to those administered 4.0 mm away from the limbus due to the anatomy of the ciliary body. Anatomically the ciliary body is thicker anteriorly near the lens and then tapers down posteriorly toward the retina. Thicker areas of the ciliary body likely contain a higher density of sensory nerve fibers, which may contribute to increased discomfort when penetrated by a needle. Thus, the patient may experience more pain at more anterior injection locations. Consistent with this hypothesis, results from this study show that patients did experience increased pain at the 3.5 mm location compared to the 4.0 mm location. However, this was not statistically significant.

### Gender on pain perception

When pain scores were stratified based on gender, there was also no significant difference between the two injection locations in females or males. However, results showed that both genders had an increased mean pain score at injection sites 3.5 mm compared to 4.0 mm away from the limbus. Further analysis with multivariable linear regression was performed, indicating that females had experienced decreased pain compared to males overall. However, this was not significant as well and participant gender did not significantly predict pain perception at either injection site. Our results are consistent with the study by Doguizi et al. [19] who also reported no significant difference in pain scores between females and males (*p* = 0.737). On the other hand, Rifkin and Schaal [17] conducted a prospective study, consisting of 60 patients randomized to receive 3 different types of anesthesia (tetraVisc, proparacaine HCl, or tetracaine HCl) before intravitreal injection to determine factors associated with patient comfort during the procedure. This study found that females reported significantly lower pain scores than males (2.96 ± 1.78 vs. 3.52 ± 2.53, *p* < 0.01) [17]. On the contrary, a few other studies reported significantly higher pain scores in females compared to males [1, 14, 16, 29]. These conflicting results suggest the relationship between gender and pain perceived may be more complex and factors contributing to pain may be multifactorial.

### Anesthesia technique

When pain scores were stratified by anesthesia technique administered during the injection process, there was no significant difference in pain experienced at the two injections sites for either anesthesia technique. Multivariable linear regression analysis showed that lidocaine 3.5% gel was associated with a slight reduction in pain scores compared to topical 4% lidocaine, but this was not significant, and anesthesia was not a significant predictor of pain. However, our study revealed that the two techniques using lidocaine gel 3.5% applied to the surface of the injection site for 5 min or the application of 2 sequential cotton tip applicators soaked in lidocaine 4% onto the injection site for a total of 2 min each time, although did not provide complete analgesia, still effectively minimized patient discomfort as demonstrated by an overall mean pain score of less than 2.5 for both techniques. These findings are also supported by Yau et al. [30], who conducted a randomized, double-blinded, prospective trial comparing anesthetic effectiveness of three topical agents, including 4% lidocaine hydrochloride pledget, 0.5% tetracaine, or 4% cocaine (+ epinephrine 1/100,000), in 93 patients with neovascular ARMD. Patients were then asked to rate their pain immediately following injection on a visual analogue scale, and results showed that there was no significant difference in mean pain score among all anesthesia groups (*p* = 0.549) [30]. Additionally, Han et al. [12] conducted a systematic review on methods of administering anesthesia for intravitreal injections and evaluated the efficacy of various techniques. Out of an initial 239 matches, 12 studies were included in the study, which included techniques such as anesthetic eye drops, gels, anesthetic soaked pledgets, and subconjunctival injections. The review revealed that no single anesthetic technique was significantly superior to the others [12]. 

### Type of anti-VEGF medication

When pain scores were stratified by anti-VEGF medication administered, there was no significant difference in pain score between the two injections locations among any of the anti-VEGF medications administered in this study. The type of anti-VEGF medications used in our study was also not found to be a statistically significant predictor of pain. Although there was a non-significant trend, aflibercept and faricimab-svoa were associated with slight reductions in pain scores compared to bevacizumab. Our results are similar to previous findings by Ertan et al. [14] who specifically conducted a study to compare pain scores of patients during intravitreal injections with various intravitreal injections of ranibizumab, alifbercept, or a dexamethasone implant. They included 162 eyes from 162 patients and also found no statistically significant difference in pain score among the injection drugs [14]. Additionally, the study by Rifkin and Schaal [17] also supported these findings and found no statistical significance between pain scores when patients were injected with bevacizumab, bevacizumab/kenalog, or ranibizumab, and there was no difference found between ranibizumab and aflibercept in the study by Öner et al. [31]. Conversely, Inaltekin et al.^15^ also conducted a study to identify factors associated with pain levels and evaluated 104 patients who received bevacizumab, aflibercept, or ranibizumab. They determined that injections with bevacizumab resulted in higher pain scores compared to aflibercept and ranibizumab (*p* < 0.001) [15]. Additionally, Bilgin et al. [16] conducted a study to compare pain scores between ranibizumab and aflibercept injections in patients who had not previously undergone intravitreal injections. They included 72 eyes from 72 patients and found that patients who received aflibercept had higher pain scores than those who received ranibizumab (*p* = 0.04) [18].

### Indication for intravitreal injections

When pain scores were stratified based on diabetic and non-diabetic indication for anti-VEGF therapy, there was no significant difference in pain scores between the two injection locations for either indication group. However, results showed slightly increased pain scores at the injection site 3.5 mm away from the limbus location compared to the site 4.0 mm away from the limbus in patients with both diabetic and non-diabetic indications. Furthermore, multivariable regression analysis was performed, revealing that diabetic and non-diabetic indications for treatment did not significantly predict pain scores either. In the past literature, several studies have demonstrated a significant correlation with pain score and the patient’s indication for anti-VEGF therapy. Segal et al. [33] conducted a study, including 225 eyes of 225 patients, to determine what factors were correlated with pain while undergoing intravitreal bevacizumab injections, and they discovered patients with DME reported significantly lower pain scores than patients with ARMD. Similarly, Inaltekin et al. [16] reported that patients with DR experienced significantly lower pain scores than patients with RVO and ARMD. In contrast, findings in Ertan et al. [14], Karimi et al. [1], Rifkin and Schaal [17], and Doguizi et al. [19] reported no significant differences in pain scores based on indication for injection.

### Injection history

Rifkin and Schaal demonstrated that average pain score decreased significantly with each subsequent intravitreal injection. The average for the first injection was 6.58 ± 1.33, followed by 4.22, ± 0.80, then 2.25 ± 0.60, and finally 1.65 ± 0.68 for the 4th injection. Karimi et al. [1] also had presented findings of a negative correlation between pain and the number of previous injections. A potential explanation for this pattern could entail decreasing anxiety levels after each injection as past studies have shown a strong correlation between anxiety and pain levels experienced during the injection [15, 32, 33]. On the contrary, when our patients were stratified based on number of previous injections in this study, results indicated that there was no significant difference in pain perceived at injection sites 3.5–4.0 mm away from the limbus in patients with a history of either 1–3 injections or greater than 3 injections. Results also showed increased pain scores at the 3.5 mm location compared to the 4.0 mm location in both injection groups, although not significant. Multivariable linear regression analysis revealed that a history of 1–3 injections compared to more than 3 injections was associated with a slight increase in pain scores, although this was not statistically significant. However, injection history was not a significant predictor of pain overall. This discrepancy may be due to the small sample size in our study that may not have had enough power to detect this difference. Our findings align with Ertan et al. [14], Inaltekin et al. [15], and Moisseiev et al. [20], in which they reported no significant correlation between pain score and number of previous injections.

### Age and ethnicity

Ertan et al. [14] found that age was negatively correlated with pain and hypothesized that those who are older may have decreased corneal nerve density and therefore may experience less pain during injections. On the contrary, Karimi et al. [1] and Inaltekin et al. [15] reported no correlation between age and pain perception while undergoing intravitreal injections. Our study aligned with the latter and showed that age was not a significant predictor of pain. One caveat Karimi et al. [1] mentioned is that as humans age there is progressive nerve density reduction in the cornea, which is particularly noticeable past the age of 70, and thus may have also affected our results since most patients in this study were older than 70 as well [1, 34]. Findings in the study by Rifkin and Schaal [17], who divided patients into groups based on age greater or less than 65, also supported this theory as their patients older than 65 reported decreased pain levels compared to patients younger than 65. Other patient demographics such as ethnicity in our study also did not significantly predict pain experienced during an injection.

### Needle gauge size

In our study, we used the same brand, 32-gauge needle to administer all intravitreal injections. Previous research examining needle gauge size has yielded conflicting results regarding its impact on pain perception. Van Asten et al. [29] investigated whether 33- versus 30-gauge needles had a significant effect on pain perception. Thirty-six patients were included in their study to receive two injections, using both needle gauges administered six weeks apart. They rated their pain using a visual analogue scale and results showed no significant difference in pain levels between the two sizes [29]. Similarly, Haas et al. [16] conducted a randomized clinical trial including 208 patients, with half receiving injections with the 30- or 27-gauge needles. Results showed that there was no significant difference in pain between the two sizes. On the other hand, the study by Güler et al. [35] found that the 27-gauge needle was significantly more painful than the 30-gauge needle. Similarly, Rodrigues et al. [36] also determined that patients had significantly increased pain from using 26- and 27-gauge compared to the thinner 29- and 30-gauge needles. A subsequent meta-analysis was performed recently by Butler et al. [13], which consisted of 9 studies with a cumulative total of 998 patients and 1004 eyes. All pain outcomes from these papers were transformed into standardized effect sizes using Cohen’s d, and they found a clear relationship between thinner needle gauge sizes and lower pain experience [13]. 

### Needle entry technique

In addition to the location of the intravitreal injection, other aspects of the needle entry that have been examined include the angle and velocity of needle insertion. Sternfeld et al. [37] performed a randomized clinical trial that included 180 patients, who were assigned to receive four different injection techniques: slow tunneled, slow straight, fast tunneled, or fast straight. The tunneled technique involved inserting the needle diagonally at a 45-degree angle into the sclera, while the straight technique was performed perpendicularly. The speed of the injection was classified as fast (0.8 s) or slow (2 s). Pain was rated on a visual analogue scale, and they found no significant difference between patients given slow injections vs. fast injections (regardless of technique) or between straight vs. tunneled (regardless of speed) [37]. We did not explore this parameter as the technique of injection was the same in all injections in our study.

## Strengths and limitations

Strengths of our study include the use of a crossover design, in which each patient could serve as their own control since each received both the injections at 3.5 mm and 4.0 mm away from the limbus, thus limiting potential confounding factors as a cause of differences in pain perception. Additionally, this was a single-blinded and randomized study, leading to reduced bias and increased objectivity and internal validity. All injections were also performed by a single physician, using a standardized injection technique, with consistent application of the same anesthetic agent for each patient and needle size. Some limitations of the study include the small sample size of 53 patients calculated for the primary outcome and may be underpowered to detect differences with comparisons within subgroups of gender, injection history, indication for injection, or medication type. A multivariable regression analysis involving numerous confounding variables typically requires a larger sample size than was available in the present study. However, the analysis was conducted under the assumption of approximate normality based on the central limit theorem as the sample size was greater than or equal to 30. Further studies should be performed with a larger sample size to confirm the results found in this trial. Another drawback includes only administering a single injection at each location. The visual analogue scale also may differ patient to patient due to individual personality or emotional state at the time of injection. The scale also does not provide details on quality or allow patients to characterize the type of pain felt.

## Conclusion

In conclusion, the findings of this study indicate that there is no statistically significant difference in pain perception between administering intravitreal injections at distances 3.5–4.0 mm away from the limbus, even when stratified based on patient demographics, history, and type of medications administered. To the best of our knowledge, this is the first study to compare two injection locations in relation to the limbus. Overall, we recommend that injection placement be determined by physician preference and experience to reduce error and complications.

## Data Availability

The datasets used and/or analyzed during the current study are available from the corresponding author on reasonable request.

## Abbreviations

AMD: Age-related macular degeneration
Anti-VEGF: Anti-vascular endothelial growth factor
CNVM: Choroidal Neovascular Membrane
CME: Cystoid Macular Edema
DME: Diabetic macular edema
DR: Diabetic retinopathy
IVI: Intravitreal injections
PDR: Proliferative diabetic retinopathy
RVO: Retinal vein occlusion
VH: Vitreous hemorrhage
